# Quantification of mitochondrial DNA copy number in suspected cancer patients by a well optimized ddPCR method

**DOI:** 10.1016/j.bdq.2017.08.001

**Published:** 2017-08-31

**Authors:** Ashfaque A. Memon, Bengt Zöller, Anna Hedelius, Xiao Wang, Emelie Stenman, Jan Sundquist, Kristina Sundquist

**Affiliations:** Center for Primary Health Care Research, Lund University/Region Skåne, Malmö 20502, Sweden

**Keywords:** Biomarker, Cancer, Droplet digital PCR, ddPCR, Mitochondrial DNA, mtDNA, Nuclear DNA

## Abstract

Changes in mitochondrial DNA (mtDNA) content is a useful clinical biomarker for various diseases, however results are controversial as several analytical factors can affect measurement of mtDNA. MtDNA is often quantified by taking ratio between a target mitochondrial gene and a reference nuclear gene (mtDNA/nDNA) using quantitative real time PCR often on two separate experiments. It measures relative levels by using external calibrator which may not be comparable across laboratories. We have developed and optimized a droplet digital PCR (ddPCR) based method for quantification of absolute copy number of both mtDNA and nDNA gene in whole blood. Finally, the role of mtDNA in suspected cancer patients referred to a cancer diagnostic center was investigated.

Analytical factors which can result in false quantification of mtDNA have been optimized and both target and reference have been quantified simultaneously with intra- and inter-assay coefficient variances as 3.1% and 4.2% respectively. Quantification of mtDNA show that compared to controls, solid tumors (but not hematologic malignancies) and other diseases had significantly lower copy number of mtDNA. Higher mtDNA (highest quartile) was associated with a significantly lower risk of both solid tumors and other diseases, independent of age and sex. Receiver operating curve demonstrated that mtDNA levels could differentiate controls from patients with solid tumors and other diseases.

Quantification of mtDNA by a well optimized ddPCR method showed that its depletion may be a hallmark of general illness and can be used to stratify healthy individuals from patients diagnosed with cancer and other chronic diseases.

## 1 Introduction

Mitochondria have their own genome and contain the only non-chromosomal DNA in humans and its role in a variety of homeostatic and signaling processes is well established [1], [2]. The mitochondrial genome contains double stranded 16.6-kb circular unmethylated DNA [3] and unlike nuclear genome, which contains only two copies per cell, it contains multiple copies per cell, depending on cell type and origin [4]. Mitochondrial DNA (mtDNA) is highly susceptible to oxidative stress, due to its close proximity with high concentration of reactive oxidative species (ROS) produced in mitochondrial matrix, which may lead to mitochondrial dysfunction, characterized by a loss of efficiency in the electron transport chain and reduction in energy production [5]. Mitochondrial dysfunction can affect cellular functions and results in a variety of human diseases such as cancer [6], [7], neurodegenerative diseases [8], [9], cardiovascular diseases [7], [10], diabetes and metabolic syndrome [11], [12], autoimmune diseases [13], [14], neurobehavioral and psychiatric diseases [15], [16], chronic fatigue [17], gastrointestinal disorders [18], musculoskeletal diseases [19] and chronic infections [20], however results are controversial. Specific signatures of mitochondrial dysfunction, such as variations in copy number are associated with disease pathogenesis and/or progression are becoming increasingly important [21]. Changes in mtDNA content may be useful as clinical biomarkers for multiple diseases but their use can be limited because of several analytical factors affecting quantification of mtDNA copy number. MtDNA content has been analyzed by conventional Southern-blot hybridization method which requires large amount of sample, is laborious and is semi quantitative [22]. More recently quantitative real-time PCR (qPCR) method has been used to measure mtDNA content. More than 97% of mitochondrial genome is duplicated in nuclear genome (pseudogenes, also known as nuclear insertions of mitochondrial origin) and can be co-amplified by the primers targeting mitochondrial genome [23]. Therefore, measuring ratio between mtDNA and nuclear DNA (nDNA) has been method of choice for mtDNA quantification [24]. However, the use of external calibrators is the core for analytical performance in qPCR which may vary among laboratories. Moreover, because of structural differences in mtDNA and nDNA various factors from DNA extraction to quantification can affect the final results [25], suggesting that new and well optimized methods are needed for accurate mtDNA quantification. Droplet digital PCR (ddPCR) is a high-throughput method that is based on water-oil emulsion droplet technology and uses reagents and workflows similar to the qPCR, however, in ddPCR, a sample is fractionated into thousands of droplets and PCR amplification of the template molecules occurs in each individual droplet which reduces bias from amplification efficiency and PCR inhibitors. It provides absolute quantification (absolute copy number) of the samples in question and does not require external calibrators [26], [27]. Furthermore, compared to real-time PCR, ddPCR showed greater precision (coefficients of variation decreased by 37–86%) and improved day-to-day reproducibility (by a factor of seven) [28].

The major aim of the study was to develop a well optimized ddPCR based method for simultaneous quantification of absolute copy number of both mtDNA and nDNA by taking into account major analytical factor which may potentially affect the quantification of mtDNA copy number. We also aimed to investigate the role of mtDNA copy number variations in cancer and other diseases and its role as a potential diagnostic marker for these diseases.

## 2 Material and methods

### 2.1 Study population

In October 2012 a project was initiated in primary health care in Regions Skåne for fast track diagnosis of patients with suspected cancers: a diagnostic center (DC) for early detection and better prognosis for cancer patients. The DC is located at the Central Hospital in Kristianstad as an independent unit within the medical clinic. It offers investigations with very short waiting times (<22 days) for patients suspected of having cancer. The project was initiated by Region Skåne and the southern regional cancer center (RCC Syd).

The primary health care centers (PHCCs) were invited to refer patients that were 18 years or older with one or more of the following symptoms: fatigue, weight loss more than 5 kg, pain/joint pain, prolonged fever, unexplained pathological lab values e.g. erythrocyte sedimentation rate, serum alkaline phosphatase, serum calcium, anemia, or suspected metastasis.

When a baseline investigation at the PHCC could not explain the symptoms, patient with suspected cancer was referred to the DC and were objectively evaluated. Whole blood samples from controls and patients were collected in ethylenediamine tetraacetic acid (EDTA) containing tubes and within 4 h were stored at −80 °C for later use. The study was performed according to the Declaration of Helsinki. The regional ethical committee at Lund University approved the study (approval no. 2012/449) and written informed consent was given by all the participants in the study after full explanation of the purpose and nature of all procedures.

### 2.2 Droplet digital PCR

Droplet digital PCR (ddPCR) was performed in investigator's laboratory. Total genomic DNA was extracted from whole blood (200 μL) using QIAamp 96 DNA Blood (Qiagen, Inc., Hilden, Germany) according to manufacturer’s instructions. DNA concentration was quantified by Thermo Scientific™ NanoDrop 2000 and 1 ng of DNA extract was loaded in the final reaction after performing serial dilution for both mtDNA and nDNA as described below. Extracted DNA was either frozen at −20 °C for long-term storage, or kept in the fridge at around 4 °C for usage within days. In this study, ddPCR system included automated droplet generator and reader from Bio-rad, (QX200 Droplet Digital PCR, Bio-rad, Hercules, California, USA), which fractionates samples into ∼20,000 droplets. For mtDNA quantification, two sets of primers and probes targeting the mitochondrially encoded NADH dehydrogenase 1 (*MT-ND1)* and mitochondrially encoded NADH dehydrogenase 6 (*MT-ND6)* genes and for nDNA quantification, two sets of primers and probes targeting the eukaryotic translation initiation factor 2C, 1 (*EIF2C1)* also known as argonaute 1, RISC catalytic component (Gene ID: 26523) and ribonuclease P/MRP 30 kDa subunit (*RPP30),* were selected for quantification of absolute copy number by ddPCR. All primer and probes were obtained from Bio-rad (Hercules, California, USA). Sequence and other information about primers and probes is available at www.bio-rad.com with following ID numbers: MT-ND1 (assay ID: dHsaCPE5029120, sequence accession number: NC_012920.1 and context sequence: CTCTAGCCTAGCCGTTTACTCAATCCTCTGATCAGGGTGAGCATCAAACTCAAACTACGCCCTGATCGGCGCACTGCGAGCAGTAGCCCAAACAATCTCATATGAAGTCACCCTAGCCATCATTCTACTATCAACATTACTAATAA), MT-ND6 (assay ID: dHsaCPE5043480, sequence accession number: NC_012920.1 and context sequence: CACCAATAGGATCCTCCCGAATCAACCCTGACCCCTCTCCTTCATAAATTATTCAGCTTCCTACACTATTAAAGTTTACCACAACCACCACCCCATCATACTCTTTCACCCACAGCACCAATCCTACCTCCAT), EIF2C1 (assay ID: dHsaCP1000002, sequence accession number: NM_012199.2 and context sequence; TGGTTCGGCTTTCACCAGTCTGTGCGCCCTGCCATGTGGAAGATG ATGCTCAACATTGATGGTGAGTGGGGAGAGCTATGGAGCCAGGG GCACCCCAAGTCCAGTGACCACACTCCCAGCCTC) and RPP30 (assay ID: dHsaCP1000485, sequence accession number: NM_006413.4:NM_001104546.1 and context sequence; TTAAGTAACTTGTAAGTGGTAGTGCATAGACTTTAAATCAGGCAGA CTGACACTAGAGTTCACATTCATAACCACTCCTCAAATGTCCTCCT ACTCTTGACATCTAGACTCAGGATGGACCTG). Probes targeting mtDNA were attached with FAM fluorophore whereas nDNA targeting probes were attached with HEX and had lowa Black^®^ FQ quencher on all probes. DdPCR method was performed according to manufacturer’s instructions with some modifications as described below. First amplification was performed in a 20 μL multiplex reaction containing 1 ng of purified DNA from whole blood, 900 nM of primers and 250 nM of probes, 2X ddPCR Supermix for probes (no UTP) and 5U/reaction HindIII enzyme (Thermo Scientific, Hudson, NH, USA). The PCR plate was sealed with an automated sealer and was incubated for 20 min at room temperature to allow digestion with restriction enzyme (HindIII). Samples were subjected to droplet generation by an automated droplet generator and later end-point PCR was performed. Cycling steps for the ddPCR were as follows: initially an enzyme activation at 95° C for 10 min (1 cycle) followed by 40 cycles of denaturation and annealing (each cycle at 94° C for 30 s and 60° C for 1 min) and finally enzyme deactivation at 98° C for 10 min (1 cycle). The PCR plate was incubated overnight at 4 °C. This additional step significantly improved the droplet recovery to maximum (19,000–20,000 droplets). Finally droplets were read on droplet reader and data were analyzed using QuantaSoft™ Software which determines the numbers of droplets that were positive and negative for each fluorophore in each sample. The fraction of positive droplets was then fitted to a Poisson distribution in QuantaSoft™ Software to determine the absolute copy number in units of copies/μl. DNA preparation and PCR experiments were performed in separate designated rooms and each run included negative and positive controls. No significant hazards or risks are associated with the reported work.

### 2.3 Statistics

Characteristics of the participants and mtDNA levels in each group are presented in Table 1 and were compared by Pearson chi-square test (dichotomized variables) or by two tailed t- test (continuous variables). Linear regression analysis was performed to adjust mtDNA levels with age (Table 1). Linearity of the assays was tested by linear regression and R-square (R^2^) was calculated for best fit. MtDNA levels were normally distributed and comparisons of mtDNA levels between most common disease groups and controls were performed by two-tailed *t*-test. Odds ratios were calculated by univariate and multivariate logistic regression analysis adjusted for age and sex. We used receiver-operating characteristic (ROC) curves to analyze the diagnostic potential of the mtDNA for solid tumors and other diseases. The accuracy is measured by the area under the curve (AUC). An AUC value of 1 represents a perfect test while an AUC of 0.5 indicates no predictive power of the test. AUC values and 95% confidence intervals (CI) for each group are presented. Statistical analyses were carried out in SPSS software version 23 (IBM, Armonk, NY, USA). Graphs and figures were prepared in GraphPad prism software version 7.

**Table 1:** Characteristics and mtDNA levels (mtDNA/nDNA copies/μL) in DC samples and healthy donors.

Classification	Age Median (min-max.)	Gender %Men/Women	MtDNA Mean ± SD	p-value	Adjusted p-value^b^
Controls (All)	48 (20–87)	57/43	136 (30)		
Healthy donors	44 (20–67)	60/40	136 (30)	0.65*	
Healthy at DC	66 (24–87)	48/52	139 (29)		
Cancer (All)	71 (20–89)	59/41	113 (34)	<0.0001^a^	<0.0001^a^
Hematologic	71 (52–82)	56/44	124 (48)	0.13^a^	
Solid	71 (20–89)	56/44	109 (28)	<0.0001^a^	
Lung	71 (61–86)	69/31	109 (26)	0.004^a^	
Colon	72 (57–83)	38/62	94 (24)	0.001^a^	
Urogenital	69 (51–79)	79/22	100 (24)	0.002^a^	
Others	73 (20–89)	57/43	117 (21)	0.001^a^	
Other diseases (All)	68 (18–90)	46/54	115 (25)	<0.0001^a^	<0.0001^a^
Autoimmune	70 (31–86)	58/42	113 (25)	<0.0001^a^	
Musculoskeletal	68 (53–87)	35/66	116 (18)	<0.0001^a^	
Infectious	64 (33–90)	57/43	113 (26)	<0.0001^a^	
Gastrointestinal	69 (31–84)	31/69	120 (23)	0.005^a^	
Anemia	70 (21–86)	22/78	116 (23)	0.007 ^a^	
Psychiatry	50 (21–70)	67/33	119 (32)	0.16^a^	
Neurological	65 (55–79)	60/40	102 (09)	<0.0001^a^	
COPD	72 (59–86)	80/20	109 (20)	0.03^a^	
Others	70 (18–87)	44/56	117 (30)	0.002^a^	

Healthy at DC; patients were assessed as healthy. COPD; Chronic obstructive pulmonary disease.

*mtDNA levels in healthy donors vs healthy at DC. ^a^ mtDNA levels in all controls vs disease. ^b^Adjusted with age.

## 3 Results

### 3.1 Selection of reference (nDNA) and target genes (mtDNA)

Different DNA extraction methods has been employed with variable results, however comparison of extraction kits show that QIAmp DNA kit, used in the study, was the most precise extraction kit with a coefficient of variation of 5.7% [25]. Furthermore, whole blood samples collected in EDTA tubes were used for mtDNA quantification as it gives a higher yield than the buffy coat samples, as previously reported [25]. Copy number variation (CNV) assays targeting two mtDNA (target) genes (*MT-ND1* and *MT-ND6*) and two assays targeting nDNA (reference) genes (*EIF2C1* and *RPP30*) were used in this study for absolute quantification of mtDNA and nDNA. *RPP30* has previously been used as a reference gene [29] and *EIF2C1* is located at a highly conserved region of the chromosome 1 [30]. However, to investigate their role as a suitable reference in our cohort, stability of reference genes was checked by quantifying the nDNA copy number in all samples and both assays showed single copy for both targets and no amplification or deletion was found in our samples. An example of *EIF2C1* is shown in Supplementary Fig. 1A. MtDNA copy number was analyzed by the *MT-ND1* and *MT-ND6*. As most of the mitochondrial genome is duplicated in the nuclear genome we run the NCBI nucleotide BLAST^®^ search using blastn and found no similarities between the mtDNA target sequences with the nuclear genome. Furthermore, we also compared the mtDNA levels quantified by both mtDNA target genes using the same reference gene (*EIF2C1*). MtDNA quantified as ratios between MT-ND1/EIF2C1 or between MT-ND6/EIFC1 showed a strong correlation, suggesting that both assays were equally suitable for mtDNA quantification (Supplementary Fig. 1B). Based on these results, MT-NDI (target) and EIF2C1 (reference) were used for further analysis.

### 3.2 Enzymatic digestion

**Fig. 1:**
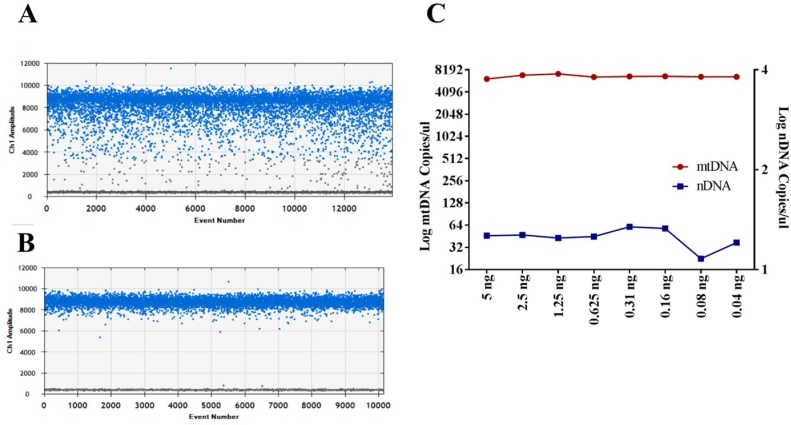
Droplets separation before (**A**) and after (**B**) enzymatic digestion. Levels of both mtDNA (red line) and nDNA (blue line) quantified in serially diluted DNA samples (n = 1 for each dilution). Data are corrected for dilution factor and are representative of two replicated experiments (**C**).

Tandem genes can have an effect on analysis of copy number and enzymatic digestion has been suggested to eliminate this effect. Prior to ddPCR, genomic DNA was digested with a restriction enzyme. Sequence of interest was checked for various enzymes using RestrictionMapper (http://www.restrictionmapper.org) and it was found that HindIII did not digest the sequences of interest and therefore all samples were digested with HindIII prior to PCR. Interestingly, digestion of DNA also improved the separation of positive droplets from negative droplets in our assay (Fig. 1A and B). Dilution error is another issue which can affect the final results [31] and we investigated it by performing serial dilutions of both mtDNA and nDNA. We could not detect any dilution error even at a very low mtDNA copy number. However, nDNA showed slight variation only at a very low DNA loading concentration (0.08 ng DNA loading concentration) (Fig. 1C) and was most likely due lower efficiency of the assay at this very low concentration of the DNA extract rather than the any dilution error. Additional sonication step did not have any effect on the results (data not shown).

### 3.3 Optimal concentration and linearity

**Fig. 2:**
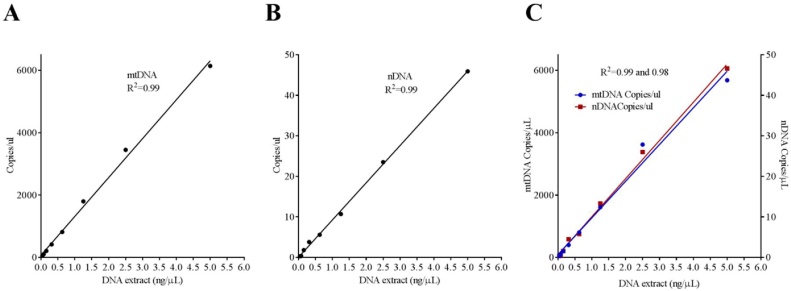
Linearity of the mtDNA (**A**) and nDNA (**B**) as single-plex and as multiplex assay (**C**) is shown in serially diluted samples. R^2^ for goodness of the fit is shown. Results are representative of two replicated experiments.

Published studies using qPCR use variable loading concentration (10–20 ng**)** of DNA extract for quantification of both mtDNA and nDNA without considering linearity of the assay. It is particularly important to load optimal DNA load especially when mtDNA copies are known to be significantly higher compared to nDNA. In our assay, we found that 10–20 ng of DNA extract was very high and resulted in overloading i.e. no negative droplets were left for quantification. We, therefore, performed a serial dilution of DNA load (5 ng, 2.5, 1.25, 0.625, 0.31, 0.16, 0.08, 0.04 ng) and linearity for both mtDNA and nDNA was checked by dilution curve. We found a strong linearity for both mtDNA and nDNA (Fig. 2A and B, R^2^ = 0.99) and therefore suggest 1–5 ng as optimal concentration of DNA extract depending on the sample type and disease in question. In this study we used 1 ng of DNA extract for quantification of both mtDNA and nDNA as an optimal concentration for simultaneous quantification of both targets as described below.

### 3.4 Multiplexing

Finally, both mtDNA and nDNA were multiplexed and run on serially diluted samples as mentioned above and their performance was compared with their corresponding single-plex assays. We found similar efficiency of mtDNA and nDNA quantified simultaneously in the same well as their corresponding single-plex assay (Fig. 2C, R^2^ = 0.99 and 0.98). All assays were included with two positive (with known copies of mtDNA and nDNA) and two negative controls (DNase free water). No amplification was found in negative controls. Intra- and inter-assay coefficient variances were calculated as 3.1% and 4.2% respectively. Intra-assay coefficient variances were calculated by quantifying replicates of samples with known mtDNA levels (high mtDNA, n = 4 and low mtDNA, n = 4) in the same plate. Inter-assay coefficient variances were calculated by samples (high and low) pipetted on each plate (n = 5) run on different days. All samples were analyzed by multiplex assay and results are presented as ratio between absolute copy numbers of mtDNA and nDNA (mtDNA/nDNA copies/μL) as described below.

### 3.5 Quantification of mtDNA in cancer and other diseases

As of December 2015, 393 consecutive suspected cancer patients were referred to the DC. 103 patients were excluded because of following reasons; declined participation (n = 23), severe psychological disorders or dementia or too ill for outpatient investigation (n = 38), patients did not fulfilled referral criteria, as mentioned above (n = 17), patients did not speak Swedish (n = 15) and referral from other than the primary health care center (n = 10). Remaining 290 patients were objectively evaluated at the DC and 286 had blood samples available for mtDNA quantification. Of 290 consecutive patients objectively investigated at DC, 64 patients were diagnosed with cancer (solid tumors, n = 48 and hematological malignancies, n = 16), 195 with other diseases [Autoimmune disease, n = 36; musculoskeletal, n = 29; infectious diseases, n = 28; gastrointestinal, n = 26; iron deficiency anemia (n = 18), psychiatry, n = 9; neurological, n = 5; chronic obstructive pulmonary disease, n = 5 and others, n = 39)] and 27 were assessed as healthy (Supplementary Fig. 2). Patients’ characteristics and levels of mtDNA quantified in 286 samples from DC and 109 healthy blood donors are presented in Table 1 as ratio between copies/μL of target and reference genes. MtDNA levels were not significantly different between healthy blood donors and patients assessed as healthy (diagnosed with no disease) at the DC (Mean ± SD; 136 ± 30 vs 139 ± 29 respectively, p = 0.65), therefore in further analyses both these groups were combined and is called as controls in the following sections. Of the controls, 78 (57%) were males and 58 (43%) were females with median age of 47 (minimum-maximum, 20–87) and 47 (20–80) respectively. Of the cancer patients, 38 (59%) were males and 26 (41%) were females with median age of 71 (20–89) and 73 (61–89) respectively, finally of the patients diagnosed with other diseases, 89 were males (46%) and 106 (54%) were females with the median age of 68 (21–86) and 69 (18–90) respectively (Table 1). No significant difference in distribution of gender was found between control group and patients diagnosed with diseases (Chi square test for trend, p = 0.8). However, healthy donors were younger [Median age (minimum-maximum, 48 (20–87)], compared to patients diagnosed with cancer [71 (20–89), p < 0.0001] other diseases [68 (18–90), p < 0.0001], Table 1. MtDNA was negatively associated with age (Pearson correlation coefficient, ρ= −0.31, p = <0.0001) and as the controls were younger than the disease group we performed a linear regression analysis and adjusted results with age and found that compared to controls, lower levels of mtDNA in cancer and other diseases were independent of age (Table 1, see adjusted *p*-values). Furthermore, we also compared the mtDNA levels (control vs disease) in different age brackets and found that the difference between control vs. disease patients was significant throughout the different age brackets, with the exception of the 18–30 age bracket (Supplementary Table 1).

**Fig. 3:**
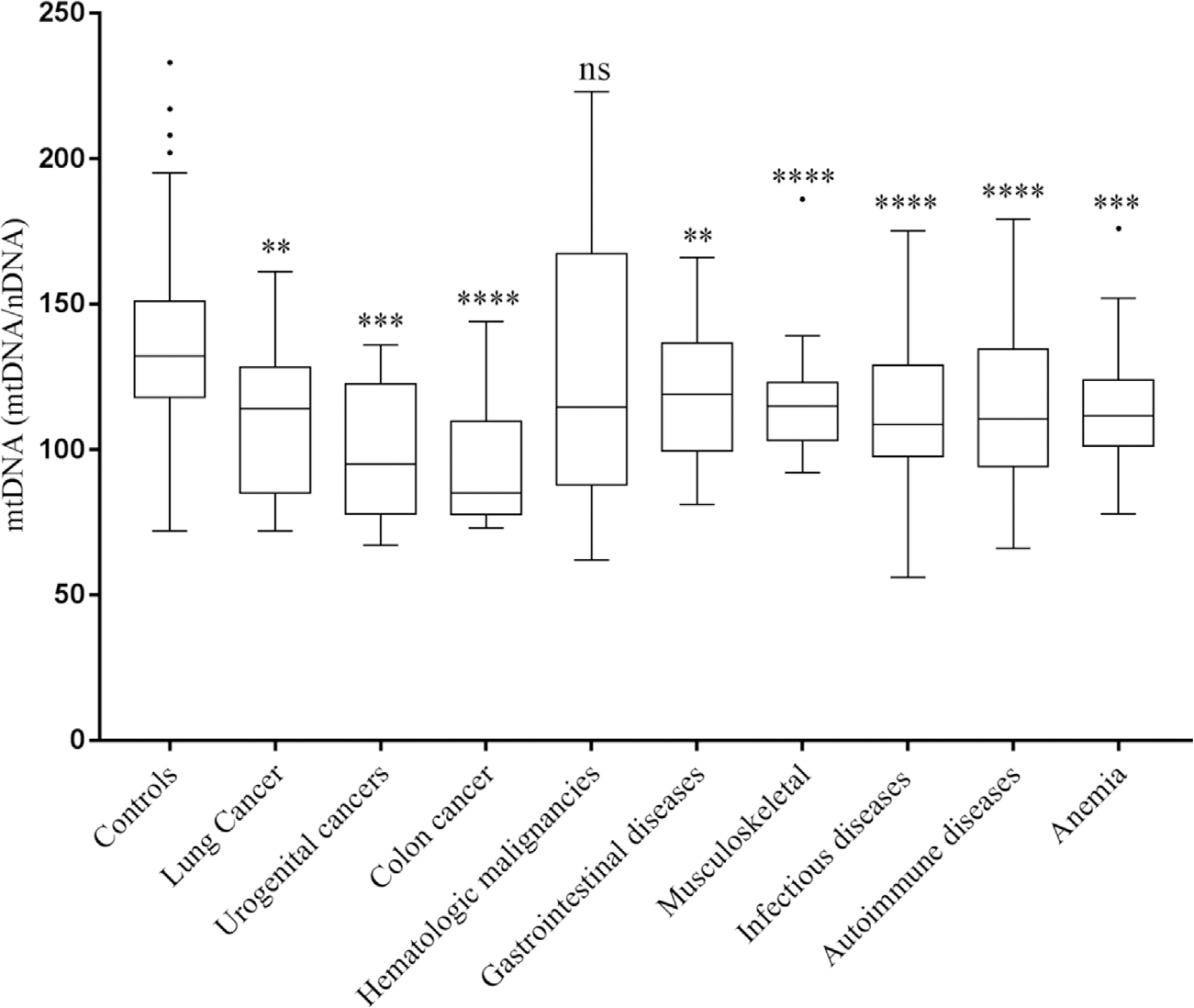
Tukey box and whiskers plot showing the levels of mtDNA in controls and the most common cancers and the most common other diseases diagnosed at the DC center are shown. The line between boxes indicates the median value, while the outer boxes represent the 25th and the 75th percentiles and whiskers show the non-outlier range. P-values calculated by tow tailed *t*-test and are shown as **<0.005, ***<0.0005 and ****<0.0001, ns = non-significant.

Levels of mtDNA of the most common cancers and other diseases are shown in Fig. 3. Most common solid cancers diagnosed at DC were lung (n = 12), urogenital (n = 9) and colon (n = 8) and among hematologic cancer, chronic lymphatic leukemia (n = 3), multiple myeloma (n = 2) and myelodysplastic syndrome (n = 2) were the most common malignancies diagnosed at the DC center. Compared to controls (mean ± SD; 136 ± 27), lung (109 ± 26, p = 0.002), colon (94 ± 24, p < 0.0001) and urogenital cancers (94 ± 24, p = 0.0003) had significantly lower levels of mtDNA copy number. Hematologic malignancies, had highly variable levels of mtDNA and were not significantly different from controls samples (124 ± 48, p = 0.13), Fig. 3. Among hematological cancers, leukemia had the highest levels of mtDNA (177 ± 48). MtDNA levels in all hematological cancers as a group are shown in Fig. 3. Among other diseases, mtDNA levels in gastrointestinal (120 ± 23, p = 0.005), musculoskeletal (116 ± 18, p < 0.0001), infectious (113 ± 26, p < 0.0001), autoimmune (115 ± 25, p < 0.0001) diseases and in iron deficiency anemia (116 ± 23, p = 0.007) were significantly lower compared to controls (Fig. 3).

### 3.6 MtDNA and risk of cancer and other diseases

**Fig. 4:**
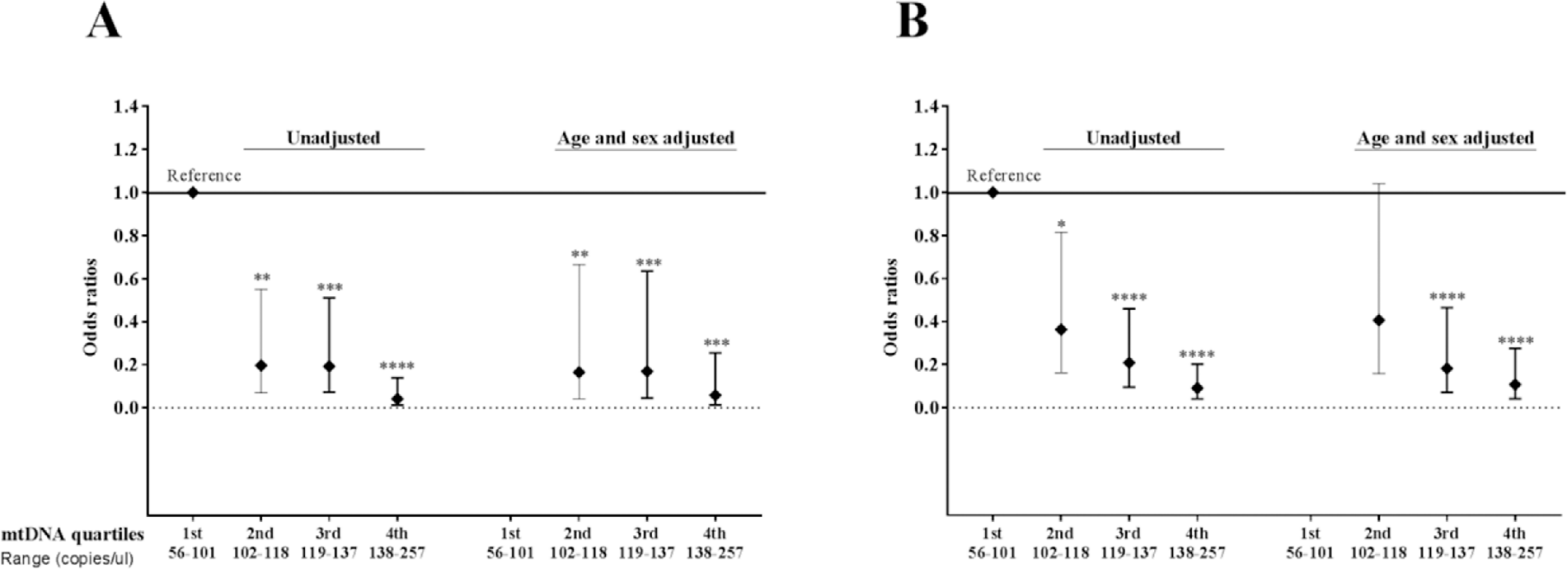
Odds ratios calculated for each quartile of mtDNA levels (shown on x-axis) in solid tumors (A) and other diseases (B). Horizontal line represents the lowest quartile as a reference. P-values calculated by univariate and multivariate (adjusted for age and sex) logistic regression analysis and are shown as *<0.05, **<0.005, ***<0.0005 and ****<0.0001.

MtDNA levels were categorized into quartiles and univariate logistic regression analysis showed that higher levels of mtDNA were significantly associated with lower risk of solid tumors in a dose dependent manner: [lowest quartile (reference) vs 2nd [Odds ratio (lower and upper 95% CI); 0.2 (0.1–0.6)] vs 3rd [0.2 (0.1–0.5)] and vs highest quartile [0.04 (0.01–0.14)], Fig. 4A. Similarly, higher levels of mtDNA were also associated with lower risk of other diseases in a dose dependent manner: (lowest quartile (reference) vs 2nd [Odds ratio (lower and upper 95% CI); 0.4 (0.2–0.8)] vs 3rd [0.2 (0.1–0.5)] and vs highest quartile [0.1 (0.04–0.2)], Fig. 4B. Multivariate logistic regression analysis adjusted with age and sex also showed that higher mtDNA levels were significantly associated with lower risk of solid tumors: lowest quartile (reference) vs 2nd [Odds ratio (lower and upper 95% CI); 0.2 (0.04–0.7)] vs 3rd [0.2 (0.05–0.6)] and vs highest quartile [0.06 (0.01–0.3)], Fig. 4A. In other diseases, except for 2nd quartile [Odds ratio (lower and upper 95% CI); 0.4 (0.2–1.0)], 3rd [0.2 (0.07–0.5)] and highest quartiles [0.1 (0.04–0.3)] were significantly associated with lower risk of other diseases, independent of age and sex, Fig. 4B). Unfortunately information on grading and staging of cancers was only available on patients who had metastatic cancer therefore we were unable to adjust it with these variables. However, we compared mtDNA in metastatic (Mean ± SD; 106 ± 28) vs non-metastatic (108 ± 18) and found no significant difference in mtDNA levels (p = 0.7), (Data not shown). Finally, mtDNA levels were not significantly associated with the risk of hematologic malignancies (Data not shown).

### 3.7 Diagnostic potential of mtDNA for cancer and other diseases

To test whether the mtDNA levels were able to separate solid tumors and other diseases from control group, receiver operating characteristic (ROC) curves and area under curves (AUC) were calculated. MtDNA levels could significantly separate solid tumors from controls (AUC = 0.77, p < 0.0001) and other diseases from control (AUC = 0.72, p < 0.0001) but not solid tumors from other diseases (AUC = 0.59, p > 0.05), Table 2.

**Table 2:** Values of area under curve and 95% confidence intervals for mtDNA in solid tumors and other diseases .

Groups	AUC	95% CI	p-value*
Solid tumors vs control	0.77	0.7−0.84	<0.0001
Other diseases vs control	0.72	0.7−0.78	<0.0001
Solid tumors vs other diseases	0.59	0.5−0.69	ns

* p-value; ROC curve analysis comparing different groups. ns; non-significant.

## 4 Discussion

Absolute mtDNA copy number is quantified by a well optimized ddPCR method in whole blood and its role in suspected cancer patients is investigated. Compared to healthy controls, mtDNA content was significantly lower in solid cancer types and other diseases. Lower levels of mtDNA were significantly associated with higher risk of solid cancers and other diseases independent of age and sex. Finally, mtDNA content could differentiate healthy controls from cancer and other diseases but not cancer from other diseases.

Mitochondrial dysfunction is known to be a hallmark of broad ranging pathologies and mtDNA content is used as a biomarker of mitochondrial dysfunction. MtDNA levels have been an attractive non-invasive marker for various diseases and recently there has been an increasing focus on evaluating mtDNA copy number in peripheral blood cell in cancers and other diseases; however conflicting results have been published [32], [33], [34]. At present, qPCR based method is used to quantify mtDNA levels by calculating a ratio between mitochondrial encoded gene DNA (target) and nuclear encoded gene (reference). This method generally quantifies relative levels of both target and reference gene on two separate experiments and requires external standards which can be different in each laboratory and makes it difficult to standardize the method for clinical use. In addition, various methodological issues have been identified which can affect the accurate quantification of mtDNA [25], [31]. The ddPCR method now represents an affordable and powerful method for absolute quantification of genetic material with performance surpassing many prevalent quantitative methods [35]. Therefore in this study we have utilized this new technology to develop a method for simultaneous quantification of target and reference by considering methodological issues which can effect correct quantification of mtDNA copy number as discussed below.

More than 97% of mitochondrial genome is duplicated in nuclear genome (pseudogenes, also known as nuclear insertions of mitochondrial origin) and can be co-amplified by the primers targeting mitochondrial genome [23]. Researchers have tried to quantify mtDNA by identifying unique regions in mitochondrial genome not duplicated in the nuclear genome [36]. However, the variation in duplication of mitochondrial genome may not be consistent across individuals and diseases. Therefore, a ratio between mitochondrial and nuclear DNA has been suggested to minimize/diminish this effect [31]. However, a selection of stable single copy reference genes is required and many studies use regions which contain repetitive and variable regions and thereby can results in wrong quantification of mtDNA [31], [37], [38] and stability of the most as reference genes is questioned [39]. Selected reference genes can vary significantly depending on sample and disease in question and there is no gold standard reference gene yet. Therefore, it is important that the stability of selected reference genes should be investigated in each study population. DdPCR provides an excellent tool to check their variability by quantifying absolute copy number of genes in study samples. We selected two reference and two mtDNA genes and investigated mtDNA copy number in our cohort by different combinations of target and reference genes and obtained highly reproducible results.

Increases in gene copy number are often the result of tandem gene duplications and DNA digestion with restriction enzymes has been suggested to efficiently separate linked copies of the target gene so that each copy is encapsulated into its own droplet and counted separately [26]. Samples were digested with restriction enzymes [40] (as described in methods section) prior to the ddPCR and interestingly, enzymatic digestion also improved the separation between positive and negative droplets. Another major issue previously reported with mtDNA quantification is the “dilution error” which is suggested to result in incorrect quantification of mtDNA [31]. Various solutions such as sonication or physical shearing of the DNA or gravimetric dilution have been recommended [27], [31], [36]. We investigated this issue by serially diluting both mtDNA and nDNA, however, did not observe the “dilution error” even at a very low mtDNA copy number. Based on our results, it seems that the variability of mtDNA quantification at the lower concentrations observed previously may be due to the structural differences in mtDNA and nDNA than the dilution error. Enzymatic digestion or sonication/physical shearing may actually linearize the mtDNA and thereby improve the efficiency of the assay.

Role of mtDNA has been investigated in variety of cancers with most studies suggesting depletion of mtDNA content in cancers [32], which is in agreement with the findings in this study. The most common cancer types in our cohort were lung and colon cancers. In agreement with our study, low levels of mtDNA were associated with lung cancer progression after chemotherapy and the degree of oxidative mtDNA damages were significantly associated with the copy number of mtDNA [41]. In another prospective study also, lower mtDNA was associated with higher risk of lung cancer and this effect was stronger in present smokers [42]. Finally, depletion of the mtDNA levels was also associated with colon cancer as shown by us and others [43]. Interestingly, in hematologic malignancies diagnosed in our cohort, especially patients diagnosed with leukemia had higher levels of mtDNA compared to solid tumors; however these levels were not significantly different from the control samples. Few studies have investigated the role of mtDNA levels in hematologic malignancies and in agreement with our study, levels of mtDNA in lymphoblastic leukemia [44] and non-Hodgkin lymphoma found to be significantly higher compared to the control group [45].

Our results show that the mtDNA depletion is not only a hallmark of cancer but of other diseases also and the most common diseases associated with lower levels of mtDNA were autoimmune, infectious, gastrointestinal, iron deficiency anemia, psychiatric and neurologic disorders etc. It was surprising to find lower levels of mtDNA in iron deficiency anemia as red cells are deficient of mitochondria. However, animal studies demonstrate that both excessive and low iron levels could result in mtDNA dysfunction and iron supplement had positive effect on mitochondrial function [46] suggesting that iron deficiency may effect mitochondrial function in other cell types containing mitochondria, however this remains to be elucidated. Overall, mtDNA depletion seems to be a biomarker of several disease processes. This makes clinical sense as virtually all tissues in the body depend to some extent on oxidative metabolism and oxidative stress may lead to mitochondrial-dysfunction or vice versa [47]. Taken together, it is possible that mtDNA dysfunction is a general phenomenon associated with the pathogenesis of multiple diseases.

A possible mechanism for mtDNA depletion in diseases can be two fold 1) accumulations of the mutations in mitochondrial genome which may lead to alterations in mitochondrial function and thereby may lead to decrease in mtDNA content [31],however the rate of this accumulation may be different in individuals depending on the genetic and environmental factors 2) unlike nuclear genome, the mitochondrial genome is intron-less, lacks histones, and has inefficient mtDNA proof-reading and DNA repair system, which renders mtDNA more susceptible to oxidative damage than nuclear genome. These factors may significantly affect the function of mitochondria and results in their dysfunction and may result in lower number of mtDNA copy number in diseases. Mutations in mitochondrial genome and their association with mitochondrial dysfunction in diseases therefore warrant further investigation.

Main strength of the study is that a well optimized ddPCR method for absolute quantification of the mtDNA is developed which can be used for reproducible and comparable results across the laboratories, as this method, unlike qPCR, does not require external standards for absolute quantification of copy number variation. Furthermore, we have investigated the role of mtDNA in multiple pathologies in a single study where patients were objectively diagnosed at the same center without inclusion biased as patients were consecutively included in the study. There were a few limitations of the study as well, for example because of the case control nature of the study we do not know whether depletion of mtDNA is the cause or consequence of the diseases. Secondly, we do not have information on tumor, node and metastasis (TNM) stages of the cancer in all patients as TNM staging was performed only in patients with metastasis and therefore we cannot conclude whether the levels of mtDNA were different in early and late stages and grades of the cancer. However, published literature strongly supports a critical role played by mtDNA content changes in contributing to cancer commencement and promotion at various stages of oncogenesis through numerous possible mechanisms [32].

## 5 Conclusions

A well optimized ddPCR method has been developed to quantify absolute copy number of mtDNA in suspected cancer patients. Low mtDNA seems to be a universal marker of broad ranging pathologies and can be used to stratify healthy individuals from patients with cancer and other diseases.

## Funding sources

This work is supported by the Swedish Research Council to Jan Sundquist, Kristina Sundquist, and Bengt Zöller. ALF funding from Region Skåne to Jan Sundquist, Kristina Sundquist and Bengt Zöller from. The Swedish Research Council for Health, Working Life and Welfare to Jan Sundquist and Kristina Sundquist. Allmänna Sjukhusets i Malmö Stiftelse för bekämpande av cancer to Ashfaque Memon.
