# Supplementary material for: Quantification of mitochondrial DNA copy number in suspected cancer patients by a well optimized ddPCR method
Source: Biomol Detect Quantif. 2017 Aug 31;13:32–9. doi: 10.1016/j.bdq.2017.08.001 (PMC5634817; doi:10.1016/j.bdq.2017.08.001)
Supplement: Supplementary file 1 [file mmc1.docx]

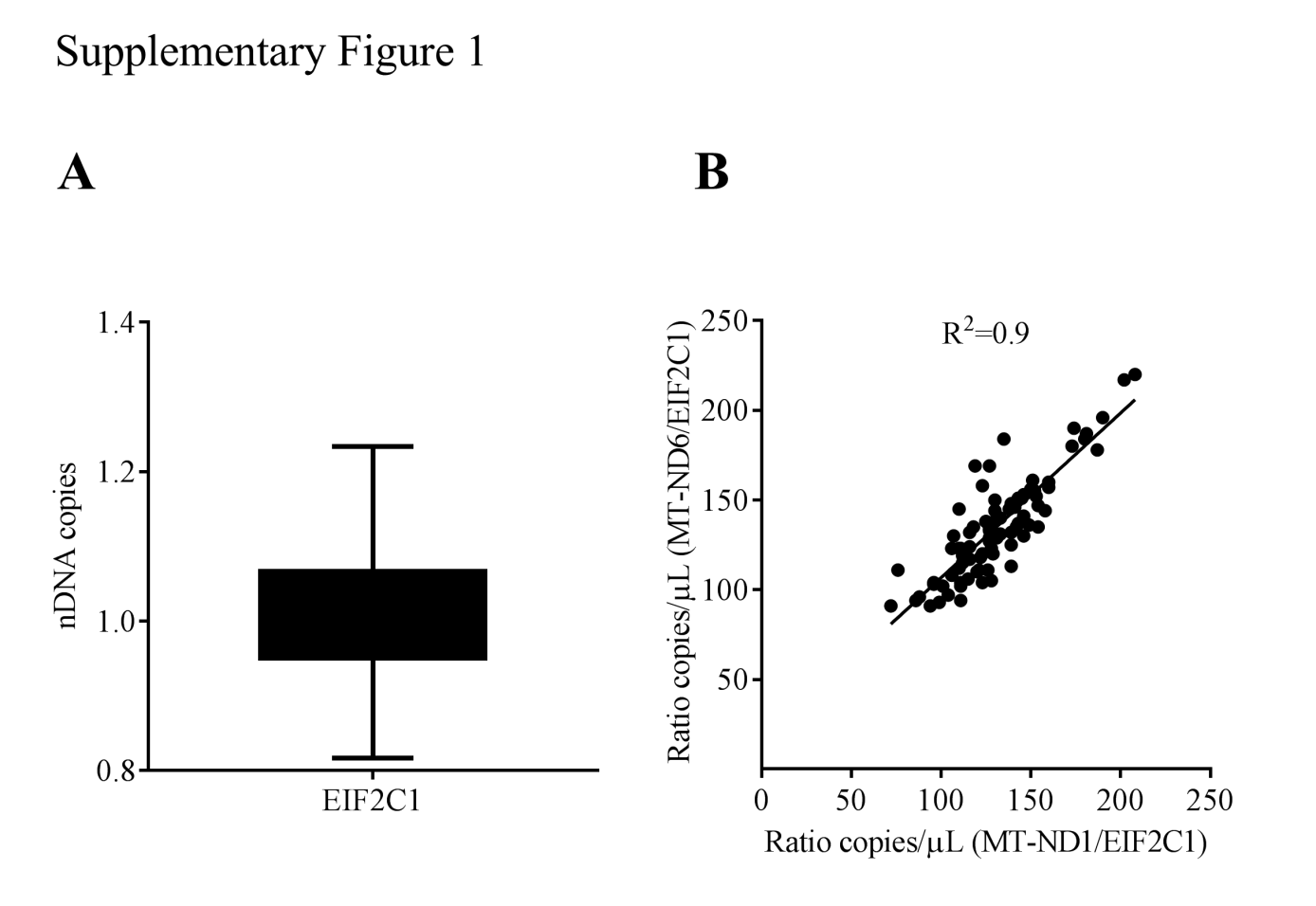


Supplementary Figure 2

Autoimmune, n=36

Musculoskeletal, n=29

Infectious, n=28

Gastrointestinal, n=26

Anemia, n=18

Psychiatry, n=9

Neurological, n=5

COPD, n=5

Others, n=39

48 were diagnosed with solid tumors

16 were diagnosed with hematologic malignancies

Lung cancer, n=13

Colon cancer, n=8

Urogenital, n=9

Others, n=18

Chronic lymphatic leukemia, n=3

Lymphomas, n=5

Others, n=8

290 patients were evaluated at DC

393 suspected cancer patients referred to DC

**103 patients were excluded from evaluation**

Declined to participate, n=23

Severe psychological disorders or dementia or too ill for investigation, n=38

Patients did not fulfilled referral criteria, n=17

Patients did not speak Swedish, n=15

Referral from other than the PHCC, n=10

64 were diagnosed with cancer

195 were diagnosed with other diseases

27 were diagnosed with no active disease

4 patients without blood samples
